# Wave intensity analysis and its application to the coronary circulation

**DOI:** 10.21542/gcsp.2017.5

**Published:** 2017-03-31

**Authors:** CJ Broyd, JE Davies, JE Escaned, A Hughes, K Parker

**Affiliations:** 1 Imperial College London, London, UK; 2 Hospital Clinico San Carlos, Madrid, Spain; 3 University College London, London, UK

**Keywords:** Wave intensity analysis, physiology, coronary vessels, diagnostic techniques, microcirculation

## Abstract

Wave intensity analysis (WIA) is a technique developed from the field of gas dynamics that is now being applied to assess cardiovascular physiology. It allows quantification of the forces acting to alter flow and pressure within a fluid system, and as such it is highly insightful in ascribing cause to dynamic blood pressure or velocity changes.

When co-incident waves arrive at the same spatial location they exert either counteracting or summative effects on flow and pressure. WIA however allows waves of different origins to be measured uninfluenced by other simultaneously arriving waves. It therefore has found particular applicability within the coronary circulation where both proximal (aortic) and distal (myocardial) ends of the coronary artery can markedly influence blood flow. Using these concepts, a repeating pattern of 6 waves has been consistently identified within the coronary arteries, 3 originating proximally and 3 distally. Each has been associated with a particular part of the cardiac cycle.

The most clinically relevant wave to date is the backward decompression wave, which causes the marked increase in coronary flow velocity observed at the start of the diastole. It has been proposed that this wave is generated by the elastic re-expansion of the intra-myocardial blood vessels that are compressed during systolic contraction. Particularly by quantifying this wave, WIA has been used to provide mechanistic and prognostic insight into a number of conditions including aortic stenosis, left ventricular hypertrophy, coronary artery disease and heart failure. It has proven itself to be highly sensitive and as such a number of novel research directions are encouraged where further insights would be beneficial.

## 1. Introduction

Wave intensity analysis (WIA) is a technique that has emerged from the field of gas dynamics and is proving useful in the assessment of coronary physiology. It is particularly applicable in this system as it can not only quantify the temporal forces acting within a single cardiac cycle, but can also separate them according to their point of origin. Unlike the systemic circulation, both distal (myocardial) and proximal (aortic) ends of the coronary system are able to exert a dynamic influence on blood flow, which can thus be independently quantified using WIA even when they occur simultaneously (Figure 3).

Waves in WIA are regarded as small wave incremental fronts, rather than the sinusoidal wavetrains used in Fourier analysis. Any arterial waveform can be created by the addition of successive wavefronts, which act to either increase or decrease pressure, and travel either forward or backward. Practically, they can be thought of as the product of the differentials of the measured pressure and flow during one sampling time at the same location within an artery. As such, the introduction of dual-tipped pressure- and flow-sensor wires has been advantageous for this field, allowing wave-intensity to be calculated using a single coronary instrument. Using this tool, 6 cyclic waves are identified per cardiac cycle with ascription to particular cardiac activities. The most clinically relevant has proven to be the backward decompression wave (BDW) which begins at the onset of ventricular relaxation and is responsible for the majority of the force drawing (or “sucking”, hence its original nomenclature^1^) blood in an antegrade direction within the coronary arteries. This wave originates from the rapid re-expansion of intramyocardial vessels that were compressed in systole creating a decompressing pressure effect. The BDW therefore provides information on the health and efficiency of the myocardium in its ability to provide its own perfusion.

Accordingly, coronary WIA has provided mechanistic and prognostic data in aortic stenosis^2^, left ventricular hypertrophy^1^, myocardial infarction,^3^ ischaemic heart disease^4^ and heart failure.^5^ Options for its employment using non-invasive methods are currently being explored which will increase its applicability to larger cohort based studies and ultimately it may be transferred more broadly into the clinical environment.

## 2. Mathematical Concepts

### 2.1. Wave intensity analysis: Conceptual theory

Although the geometry of arterial structures is complex, when dealing with wave intensity they can be regarded as one-dimensional tubes, with waves providing information on axial pressure and velocity changes. In wave intensity analysis the basic functions are incremental wavefronts. The measured pressure and flow waveforms are decomposed into the summation of successive wavefronts of different amplitudes, either positive or negative. For application to coronary studies, WIA has two advantages: it is carried out in the time domain making it easy to relate its results to particular times in the cardiac cycle and it makes no assumption about periodicity so it can be used to analyse transient conditions where Fourier analysis is invalid.

The simplest definition of a wave is a “disturbance that propagates in space and time” and invariably this propagation involves the exchange of energy. In the cardiovascular system, this exchange is between the kinetic energy of the blood and the potential energy stored in the walls of the elastic vessels. In arteries it is observed that these waves travel at a speed that is always larger than the velocity of the blood. Accordingly these waves influence the medium in which they pass in three potential ways, all of which can be assessed:

1. Direction of travel – forward or backward
2. Effect on pressure – compression or decompression
3. Effect on velocity – acceleration or deceleration

It is important to appreciate that coincident waves with opposing effects will have a summative result so that if a forward acceleration (or “compression”) wave meets a backward deceleration (or “decompression”) wave of the same magnitude there will be no net change in the velocity.

### 2.2. Wave intensity analysis: mathematical derivation

Wave intensity analysis is based on the solution of the fundamental equations for the conservation of mass and momentum in the blood vessel. The solution is based on the method of characteristics which involves rather subtle mathematics which are presented elsewhere in detail.^6^ Given the complexity of the mathematics, the results are surprisingly simple and intuitive.

The fundamental definition of wave-intensity (I) is:

\begin{eqnarray*}I=dP\,dU \end{eqnarray*}

where dP is the change in pressure and dU the change in velocity across a wave. The ‘width’ of the wavefront is implicitly defined as one sampling time.

Wave intensity has the units Wm^−2^ and its sign conveys the direction of the dominant wave such that a positive value reflects the dominance of forward-travelling waves originating proximally and negative the dominance of backward-travelling waves originating distally. A disadvantage of this definition is that its magnitude is determined by the sampling frequency. Therefore, either a universal sampling frequency needs to be adopted for standardisation or, more conveniently, the definition of wave intensity can incorporate the sampling rate as:

\begin{eqnarray*}{I}^{{^{\prime}}}= \left( \frac{dP}{dt} \right) \left( \frac{dU}{dt} \right) . \end{eqnarray*}

In this case the units are now Wm^−2^s^−1^.

The first step in calculating separated wave intensity is the observation that the pressure and velocity changes in each elemental wavefront must be related to each other for mass and momentum to be conserved. The method of characteristics solution gives these relationships, which are known as the ‘water hammer’ equations, as

\begin{eqnarray*}d{P}_{+}=\rho cd{U}_{+} \end{eqnarray*}

for forward waves (subscript ‘+’) and

\begin{eqnarray*}d{P}_{-}=-\rho c\,d{U}_{-} \end{eqnarray*}

for backward waves (subscript ‘−’). Here ρ is the density of blood and c is the wave speed.

Since our decomposition of the measured waveforms into successive incremental wavefronts assumed that the wavefronts are additive and only forward and backward waves are present, at any point at any time in the artery

\begin{eqnarray*}dP=d{P}_{+}+d{P}_{-} \end{eqnarray*}

\begin{eqnarray*}dU=d{U}_{+}+d{U}_{-}. \end{eqnarray*}

These equations and the water hammer equations give us 4 equations for the 4 unknowns, dP_+_, dP_−_, dU_+_ and dU_−_, in terms of the known changes in pressure and velocity, dP and dU.

Solving first for dP_+_

\begin{eqnarray*}d{P}_{+}=dP-d{P}_{-}=dP+\rho c\,d{U}_{-}=dP+\rho c \left( dU-d{U}_{+} \right) =dP+\rho cdU-d{P}_{+}. \end{eqnarray*}

Thus

\begin{eqnarray*}d{P}_{+}= \frac{1}{2(dP+\rho c\,dU)} . \end{eqnarray*}

Solving for dP_−_ in the same way

\begin{eqnarray*}d{P}_{-}= \frac{1}{2(dP-\rho c\,dU)} . \end{eqnarray*}

The forward and backward velocity changes follow directly from the water hammer equations

\begin{eqnarray*}d{U}_{+}= \frac{1}{2\rho c} \left( dP+\rho c\,dU \right) \end{eqnarray*}

\begin{eqnarray*}d{U}_{-}= \frac{-1}{2\rho c(dP-\rho c\,dU)} . \end{eqnarray*}

The separated wave intensities are defined in the same way as the net wave intensity so that

\begin{eqnarray*}{I}_{+}\equiv d{P}_{+}d{U}_{+}= \frac{1}{4\rho c} { \left( dP+\rho c\,dU \right) }^{2} \end{eqnarray*}

\begin{eqnarray*}{I}_{-}\equiv d{P}_{-}d{U}_{-}= \frac{-1}{4\rho c} { \left( dP-\rho c\,dU \right) }^{2}. \end{eqnarray*}

Inspection of these results shows, because of the square term, *dI*_+_ > 0 and dI_−_ < 0 which explains why wave intensity easily differentiates between forward and backward waves.

Another property of the separated wave intensity is that the forward and backward wave intensity sum to the net wave intensity, a useful result that is not immediately apparent

\begin{eqnarray*}{I}_{+}+{I}_{-}= \frac{1}{4\rho c[{ \left( dP+\rho c\,dU \right) }^{2}-{ \left( dP-\rho c\,dU \right) }^{2}]} \end{eqnarray*}

\begin{eqnarray*}= \frac{1}{4\rho c[(d{P}^{2}+2\rho c\,dPdU+{ \left( \rho c\,dU \right) }^{2})-(d{P}^{2}-2\rho c\,dPdU+(\rho c\,dU)^{2})]} =dPdU=I. \end{eqnarray*}

From these derivations we see that both the forward and backward wave intensities can easily be separated from the pressure and velocity measured in the cardiac catheterisation laboratory if the local wavespeed c is known.

Fortunately, it is possible to estimate the local wavespeed from the pressure and flow measurements. Given the inherent length of coronary arteries, a foot-to-foot approach to measuring the wave speed is not appropriate and an alternative method has been proposed and validated as well as it could be in the absence of an independent definitive measure of coronary artery wave speed.^7^ The derivation of the sum-of-squares method yields the following formula

\begin{eqnarray*}c= \frac{1}{\rho \,} \sqrt{ \frac{\sum {dP}^{2}}{\sum {dU}^{2}} } \end{eqnarray*}

where the summations must be taken over the whole cardiac cycle.

Given the uncertainties in the estimation of c, it is important to note that the net wave intensity does not require any knowledge of c, depending directly on the measured P and U, and so any diagnostic conclusions that can be drawn from the net wave intensity alone are more reliable. Quantitative calculation of the separated wave intensities, which can be particularly informative in the coronary arteries, does introduce an increased level of uncertainty into the analysis.

The wave intensity peaks can be expressed in three different but related ways (Figure 3): Firstly, the ‘peak wave intensity’ is defined as the maximum value of the wave intensity which has the units (W/m^2^) and is a measure of the flux of energy carried by the wave. Secondly, the ‘cumulative wave intensity’ is defined as the area under the wave intensity versus time curve. The cumulative wave intensity has units J/m^2^ and is a measure of the energy of the wave and accounts for the duration of the wave as well as it peak value. Finally, the ‘wave energy fraction’ is defined as the cumulative wave intensity for a particular wave divided by the integral of the wave intensity over the cardiac period. It is dimensionless and represents the fraction of the wave energy of a particular wave relative to the net wave energy generated throughout the cardiac cycle. The wave energy fraction can be useful to differentiate, for example, between changes due to a particular mechanism and changes due to an overall change of heart function.

## 3. Coronary wave intensity profile

Through the above derivation, separated wave-intensities can therefore be calculated from simultaneously acquired pressure and velocity waveforms. Within the coronary circulation historically this was achieved with individual pressure and flow sensor-tipped wires with sensors positioned at identical positions. However, with the introduction of combined, dual-tipped wires this can be done more conveniently with an individual system. Following normalisation, this wire is manoeuvred into the desired position in an approach similar to that of a conventional pressure-wire. Further manipulation may be necessary to optimise the flow signal whereupon pressure and flow is recorded. Off-line, data is digitized and collated; following which wave-intensity analysis can be calculated (Figure 2).

**Figure 1.**
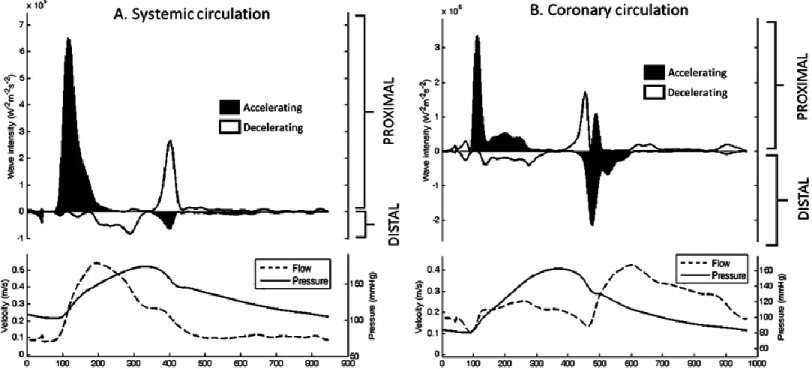
Pressure, flow and separated wave-intensity analysis obtained from the systemic (carotid artery) and coronary (left anterior descending artery) circulation. In the systemic circulation the majority of the influence from the wave-intensity profile is from the proximal end of the artery with the majority of the distal-originating waves created by ‘passive’ reflection of the proximal waves. However, in the coronary circulation there is a significant proportion of the wave-intensity profile originating distally from the ‘active’ myocardium.

**Figure 2.**
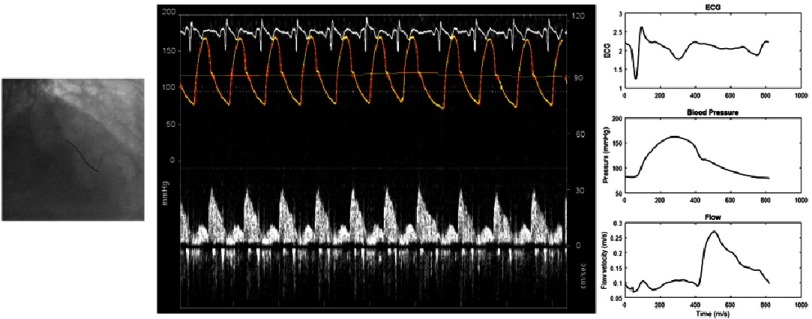
The practical construction of wave-intensity anaylsis. Following normalisation, the dual-tipped pressure and flow sensor wire is positioned appropriately (left). Further minor manipulations may be necessary to optimise the flow signal before pressure and flow are recorded (centre). This data is digitized and collated off-line to produce intra-coronary pressure and flow signals (right) which are processed to produce wave-intensity analysis as in Figure 1B.

**Figure 3.**
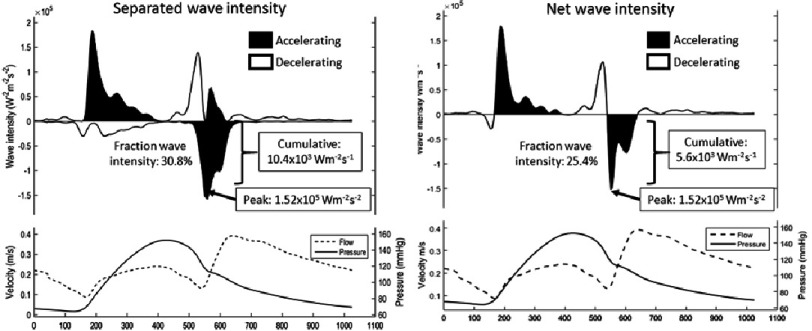
Separated and net coronary wave intensity constructed from an unobstructed left anterior descending artery. Three ways of expressive wave-intensity of the backward decompression wave are highlighted: peak, cumulative and fraction. Given the active role the myocardium plays within then coronary wave-intensity profile, the benefit of separated wave-intensity analysis is highlighted.

Initial work in patients with unobstructed coronary arteries demonstrated a repeating pattern of six waves per cardiac cycle evident in all major epicardial arteries.^1,8^ Three waves originate proximally and three distally with variable compressing/decompressing and accelerating/decelerating effects. Using the numbering system in Figure 3, the predominant waves in the coronary arteries are described below.

### 3.1. Proximally (‘aortic’) originating waves

These waves originate from the aortic end of the coronary circulation and are therefore conventionally described as ‘forward travelling’. When compressive these waves will have an accelerating effect and when decompressing are decelerating.

1.: Forward compression wave (accelerating)
  Contraction of the left ventricle with an open aortic valve results in expulsion of blood into the proximal aorta and coronary sinuses. This generates an antegrade wave, which originates in the left ventricle, is transmitted into the aorta, and down the coronary artery from proximal-to-distal.
2.: Forward decompression wave (decelerating)
  Whilst this wave originates and travels in the same direction, it has a decompressing effect resulting in deceleration. It is created from the slowing of ventricular contraction with a suction effect applied from the aortic end of the coronary artery and occurs at the end of systole.
3.: Late forward compression wave (accelerating)
  With the transition to diastole, the aortic valve closes creating a short-lived proximal-to-distal compression wave which occurs during early diastole. This has an accelerative effect on blood flow.

### 3.2. Distal (‘myocardial’) originating waves

These waves are ‘backward travelling’ (i.e., travel from distal-to-proximal) and will therefore have a reverse action to the proximally-originating waves – compression waves cause deceleration and decompression waves cause acceleration.

4.: Early backward compression wave (decelerating)
  This wave is created by the period of isovolumic contraction. In early systole, prior to the aortic valve opening, compression of the intra-myocardial blood vessels generates this decelerating compression wave.
5.: Late backward compression wave (decelerating)
  A second wave occurring in early systole, this decelerating wave is created from the summation of continuing compression of the microcirculation as well as reflection of the forward compression wave from distal reflection sites in the coronary circulation. The net effect is a distally-originating compression wave, again with a decelerating influence.
6.: Backward decompression wave (acceleration)
  The backward decompression wave occurring at the end of systole is the most clinically relevant wave. It originates from the myocardium and is decompressive, resulting in a pronounced accelerative effect. This wave is created by the re-expansion of the compressed intra-myocardial blood vessels combined with a simultaneous reduction of resistance in the myocardial microcirculation causing a sharp decrease in pressure distally and therefore a decompressive pressure gradient.

**Figure 4.**
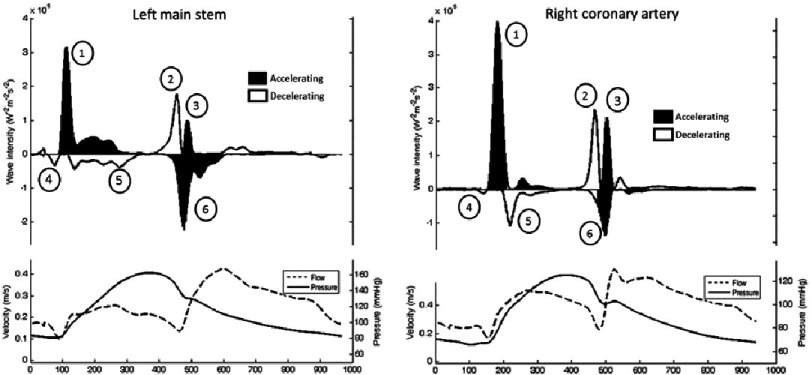
Pressure, flow and separated wave intensity analysis from the left main stem (left) and right coronary artery (right). Six waves are evident per cardiac cycle, 3 from the aortic end (1–3)and 3 from the myocardial end (4–6)of the coronary artery. All six waves are evident in both left and right coronary arteries but with differing magnitudes depending on the ventricle that is subtended. The two dominant waves are the forward compression wave (FCW –(1)) and the backward decompression wave (BDW –(6)).

This wave is evident throughout the entire coronary circulation, but in the right coronary artery a smaller BDW is seen, resulting in the observed lower diastolic velocity rates.^8^ This is probably due to the lower pressures in the right ventricle, resulting in lower intramyocardial stress in the right ventricular wall and hence a lower ‘suction’ force during diastolic relaxation (Figure 4).

The two waves which dominate in terms of magnitude are the forward compression wave (FCW) and the backward decompression wave (BDW) - both cause an accelerative force on blood flow but with differing results on actual velocity due to the presence (systole) or absence (diastole) of opposing intra-myocardial pressure.

## 4. Wave intensity in clinical studies

Whilst the six waves described above are always evident, the most clinically informative wave to date has proven to be the dominant BDW. Its terminology has varied in the literature, initially being described as a ‘suction’ wave in an effort to convey the effect it has on blood flow. As outlined above, this wave is caused by the re-expansion of the intra-myocardial blood vessels which are compressed by ventricular contraction during systole. Systolic ventricular contraction not only creates the backward compressive wave it markedly increases coronary resistance to antegrade flow. This is, in part, responsible for the failure of the forward compression wave to cause a significant increase in coronary flow velocity (similar to a car-driver trying to accelerate by simultaneously pressing both the accelerator and brake pedals). However, once myocardial relaxation begins this ‘brake’ is removed and any forces acting on blood velocity are much less opposed. Therefore, as this network of vessels re-opens, driven in part by the expanding myocardial fibres and in part through their elastic recoil, a proximal-to-distal pressure (‘decompression’) gradient is created resulting in an accelerative force and an obvious increase in coronary velocity.

Conceptually this wave could be affected by several processes. Firstly, relaxation of an increased ventricular force should cause a larger ‘suction’ effect from the myocardium resulting in a larger BDW. Secondly, if the interrogated artery had an increased density of subtended vessels the BDW would also increase. Thirdly, the efficiency with which the intra-myocardial vessels themselves re-expand will also govern the relative magnitude of the BDW. Fourthly, the efficiency with which the ventricle relaxes will also have an important effect on the BDW. These hypothesized pathophysiological influences on this wave essentially have formed the outline for the published studies to date which are examined below.

### 4.1. Left ventricular hypertrophy (LVH)

Previous large scale retrospective and prospective studies have demonstrated the unfavourable effect of left ventricular hypertrophy on prognosis independent of other cardiovascular risk factors.^9–13^ For every increment of 50g/m height in left ventricular mass the relative risk of cardiovascular disease is increased approximately 1.5-fold.^9,10^ The presence of LVH may also lead to the development of heart failure independent of other potential confounders.^14,15^ In patients who suffer an acute myocardial infarction, the presence of LVH conveys a poorer prognosis compared to those with normal left ventricular geometry^16^ and is an important contributor to the risk of sudden cardiac death in patients with coronary artery disease.^17^

At a structural level, myocardial hypertrophy leads to a distortion of myocardial architecture with additional fibrotic changes over time^10^; this disorganisation can be appreciated using conventional coronary physiology as a reflection of this adverse prognostic data. Animal models have demonstrated that the induction of LVH has a negative effect on coronary flow reserve, largely driven by an attenuated hyperaemic response.^11–13^ This effect also appears broadly reversible: in both interventional.^18–20^ and genetic^21,22^ models of LVH its reversal (with removal of the aortic-band or renal clip, or with pharmacotherapy) improves coronary flow reserve. Likewise in humans pharmacological treatment can improve CFR^23–25^ such that an 8% reduction in LV mass is associated with a 43% increase in coronary flow reserve.^17^

Wave-intensity analysis has also been employed in left ventricular hypertrophy.^1^ In a study of 20 patients with unobstructed coronary arteries a negative correlation was noted between left ventricular wall thickness and the backward decompression wave. The reason for this appears to be a disruption between the systolic compression of the microcirculation (measurable as the forward compression wave) and the resultant diastolic decompression (the backward decompression wave) evident as differences in the regression slope between these waves for septal thickness above and below 1.2 cm. Moreover, this study demonstrates the inherent strength of WIA where these subtle microcirculatory changes affecting capillary filling are revealed at rest without the need for hyperaemia.

Recently, preliminary data has recently emerged confirmed an abnormal coronary wave-intensity pattern also exists within patients with genetic hypertrophic cardiomyopathy, notably a relative reduction of the BDW in conjunction with an increase in the systolic backward-originating waves that reflect microcirculatory compression. A correlation with MRI-determined myocardial blood flow and the BDW has also been appreciated^26^ with further insights undoubtedly to appear.

There are some unanswered questions in left ventricular hypertrophy that may be assisted by further wave-intensity work. Whilst coronary flow reserve improves with the reversal of left ventricular hypertrophy there is some evidence to suggest that this process is not always complete. For example in dogs, some abnormalities persist following normalisation of left ventricular size notable with atrial pacing but not adenosine.^27^ Furthermore, in rats histological studies have shown that aortic-banding results in different changes depending on the length of time banding is present – when long-term, whilst the evident medial hypertrophy can regress following band-removal, the fibrotic changes that occur appear permanent and are accompanied by a persistent reduction in coronary flow reserve.^28^

Whilst histological studies are difficult to perform in human experiments, wave-intensity analysis may be able to play a role in determining whether these (potentially time-dependent) effects occur in humans as well. Wave-intensity is powerful enough to recognise the subtle physiological changes that reflect such histological abnormalities and as such could be used in prospective studies examining the effect of LVH therapy.

Additionally, there is some evidence that particular pharmacological agents also have differing effects on coronary haemodynamics. Studies with angiotensin-receptor antagonists have shown these agents encourage an improvement in CFR independent of LV hypertrophy^29,30^ whereas (perhaps surprisingly) some ACE-inhibitors do not.^31^ Again, (systemic) wave-intensity analysis has already shown its worth in providing further information in differing anti-hypertensive regimens.^32^ and coronary wave-intensity is an area that also would warrant exploring to that end.

### 4.2. Aortic stenosis

Disturbed coronary haemodynamics are a key part of the pathophysiology within aortic stenosis. In patients with severe aortic stenosis and angiographically normal coronary arteries, angina is reported in 30–40%^33^; this is an indicator of the presence of ischaemia in this condition and is a well-established (albeit relatively crude) marker of severity and prognosis.^34^ Microvascular dysfunction can be demonstrated in a number of increasingly sophisticated ways including lactic acid production,^35,36^ 24 hour Holter monitors,^37^ thallium scans,^38^ CFR,^33,39^ contrast echocardiography^40^ and PET scanning.^41^

Aortic stenosis has also been investigated with WIA. The first striking finding is of a dramatically increased BDW relating to the severity of the aortic lesion. This is undoubtedly due to the increased diastolic relaxation force that occurs as an elastic response to the increased systolic compression required to expel blood through a stenotic aortic valve. This effect swiftly reverses following interventional treatment of aortic stenosis with a large fall in the magnitude of the backward decompression wave.^2^ Similarly, in human subjects CFR impairment also appears to be correlated with aortic valve area and peak gradient^42^ so one can envisage that the larger the BDW at rest, the less reserve available during hyperaemia.

The ability to pace a heart with aortic stenosis and/or hypertrophy for research purposes has been explored in animal models (where abnormalities in blood flow have been demonstrated^28^) and historic studies in humans (reproducing symptoms of angina^29^). To that end, Davies *et al.* made measurements during transcatheter aortic valve intervention (TAVI) to demonstrate the effects of increasing heart rate on aortic stenosis.^43^ By increasing the ventricular pacing rate using the temporary wire required for this procedure, the BDW has been shown to decrease with increasing heart rate pre-TAVI. Most importantly, this effect is immediately reversed after valve implantation returning to what is felt to be the physiological norm.^30,31^. It has been proposed that this pre-TAVI state reflects a ‘decoupling’ of the normal ventricular-coronary mechanisms essential for maintaining normal coronary perfusion. It is likely that through this decoupling process patients begin to experience angina and that a ‘re-coupling’ is one of the reasons for the instantaneous reduction in mortality following aortic valve replacement.^44^

**Figure 5.**
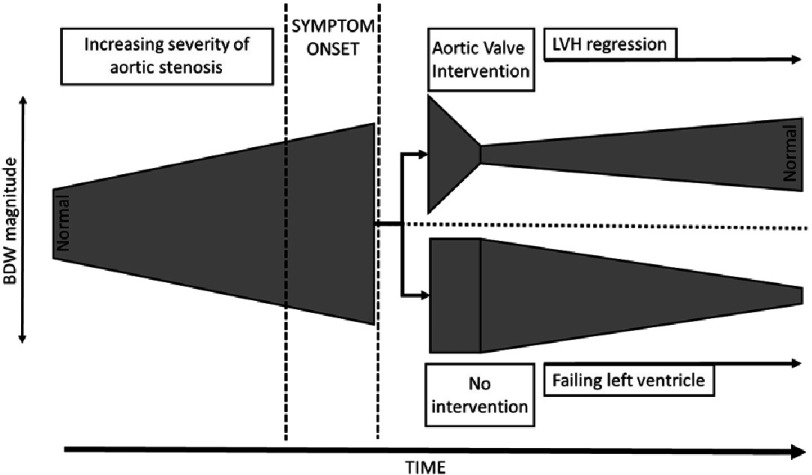
Hypothesized schematic representation of the magnitude of the backward decompression wave represented by the width of the grey region following the progression and treatment options in aortic stenosis. As the aortic valve lesion worsens the backward decompression wave increases to supra-normal levels. If intervention is performed at an appropriate time the backward decompression wave decreases to a sub-normal level as the dominant influence is from the residual left ventricular hypertrophy. Over time, this regresses and the backward decompression wave recovers. Alternatively, if the opportunity to intervene is missed, the ventricle ultimately begins to fail resulting in a steady decline in the backward decompression wave.

However, aortic stenosis represents an interesting dichotomous state where both a significant outflow tract gradient exists in parallel with significant LVH. When the valve lesion is severe, the dominant effects on haemodynamics are certainly due to the imposed haemodynamic load (rather than from the LVH) as has been shown with PET scanning,^45,46^ TOE^47,48^ and now wave-intensity.^43^ However, following valve replacement, marked hypertrophy persists and potentially has a continuing detrimental effect on coronary haemodynamics as outlined above. With previous investigative modalities, it is difficult to tease out the relative contributions of myocardial hypertrophy and the increased ventricular pressure to this pathophysiology, which is where wave intensity analysis may be able to shed some light. More specifically, whilst CFR)^49^ and subendocardial blood flow^50^ have been shown to recover after aortic valve surgery, it is not yet clear whether there is a further LVH-regression-driven improvement in coronary haemodynamics in the post-operative state. Additionally, if the opportunity to intervene is missed, the effect on the backward decompression wave and its potential for recovery is as yet unknown providing further potential investigative avenues (Figure 5).

### 4.3. Heart failure and resynchronization therapy

Cardiac resynchronisation therapy (CRT) in left bundle branch block (LBBB) has a proven positive prognostic influence on the prevention of death and the development of heart failure,^51^ implying an improvement in coronary haemodynamics. Additionally, the effect of patient-specific atrio-ventricular (AV) delay optimisation also has an important influence on ventricular and aortic interaction and resultant filling.^52^ However, the effect on coronary haemodynamics imparted by these interventions is not so well studied. Both invasive catheter studies^53^ and PET scanning techniques^54^ have failed to show a marked change in coronary flow rates at rest but the ability of wave intensity to look beyond conventional resting indices and demonstrate more subtle changes may make it a useful tool for investigating CRT further.

Using this approach, an obvious change in the wave-intensity profile has been noted with different AV pacing protocols.^5^ The BDW increases with AV optimisation producing an increased coronary velocity time integral. Additionally, in a sub-optimally paced heart no demonstrable differences in the BDW are noted when compared with an unpaced heart. Wave intensity in CRT has therefore shown the improvement in coronary haemodynamics with optimisation of pacing regimens. It has also emphasised the importance of ventricular filling and contractility on in its myocardial blood supply – when both ventricular-ventricular and atrio-ventricular pacing are optimised the resultant force acting on coronary flow is most efficient.

Recent evidence has emerged that the effect of CRT is noticeable even in patients with mild heart failure symptoms.^51^ Again, this is an area worthy of further investigation. Particularly, it would be interesting to establish if favourable changes are noted in coronary haemodynamics (particularly wave-intensity) with CRT in these patients and if these changes occur to a similar magnitude in patients with only mild symptoms.

### 4.4. Ischaemic heart disease

WIA has been used to provide insight into patients with stable and unstable coronary artery disease. In the case of stable angina, it has been employed as a powerful measure of small but dynamic changes within the coronary circulation to investigate the phenomenon of warm-up angina, the process whereby patients suffer from less anginal symptoms after resting following an initial exercise-induced angina episode. This phenomenon was first recognised 50 years ago as an increased exercise tolerance after a first episode of exercise-induced angina on treadmill testing^55^ with reduced electrocardiographic evidence of ischaemia on repeat.^56^ At this time, a series of experiments were undertaken to further elucidate this but with only limited results due to the relative crudity of available techniques.

However, one observation noted was that the warm-up phenomenon appeared to influence coronary flow (i.e., ‘supply’) rather than myocardial oxygen consumption (‘demand’).^56^ When revisited 30 years later by the application of WIA, more specific details could be noted. In an elegant series of experiments, WIA performed distal to a coronary lesion during two successive periods of exertion has shown a relative increase in the BDW with the second phase.^4^ It appears that this ‘pre-conditioning’ of the heart results in an improved cardiac-coronary interaction resulting in this improved performance. This feature interacts with other favourable ‘warm-up’ cardiovascular changes including a reduction in central aortic pressure augmentation and reduced coronary microvascular resistance resulting in a summative or even synergistic effect.

In the area of unstable coronary disease, wave-intensity analysis has been used to offer prognostication in patients who suffered from non-ST elevation myocardial infarction. Despite optimal therapy in these patients, a number go on to develop myocardial cell infarction. The integrity of the microvasculature is a key determinant of myocardial viability and tissue perfusion as demonstrated with PET^57^ or myocardial contrast echocardiography.^58^ Additionally, a number of unusual patterns of coronary flow have been acknowledged as potential markers for microvascular dysfunction.^59,60^ This stimulated the use of WIA to explore this field and examine for different predictive markers of outcome.

Of note, the BDW in the infarct related artery (measured within 48 hours of the event) correlated with both biochemical- and MRI-markers of infarct severity. There was a reasonable correlation between the forward compression wave and ejection fraction but not with the BDW perhaps implying a relative inefficiency in energy exchange between these two, similar to that seen in left ventricular hypertrophy. Additionally, the backward decompression wave was also related to left ventricular function.

Recovery of left ventricular function can be assessed by measuring improvements in wall thickening over a period of months and has been shown to correlate well with BDW values measured at the time of percutaneous treatment. Therefore it appears that WIA measured at the time of non-ST elevation infarction is both a marker of infarct size and also conveys information on myocardial integrity, an important predictor of functional recovery.^3^

There are several potential areas for future investigation within this subject. Particularly, it will be interesting to see if the effects noted within non-ST elevation MI can be applied to ST-elevation infarction and additionally if these changes ultimately convey prognostic morbidity and mortality data. Recent work involving the pressure and flow relationship of mid-to-late diastole has highlighted the importance of ventricular pressure on the microcirculation during myocardial infarction^61,62^ and the incorporation of this information in the WIA setting needs consideration, perhaps using simultaneously LV pressure measurements. Finally, there is potential for wave-intensity to guide adjuvant therapy such as intra-coronary thrombolysis if a significant microcirculatory disruption is noted from (presumed) downstream embolization as has been suggested from other microcirculatory investigative techniques.^63^

## 5. Conclusion

Wave intensity analysis has emerged as a powerful tool to investigate the coronary system. Six waves have been noted but at present the most clinically relevant is the backward decompression wave (BDW), which relates to the function of the distal intra-myocardial blood vessels in response to the myocardial relaxation. Whilst it is calculated from resting pressure and velocity signals its synergistic strength in recognising subtle abnormalities that are otherwise imperceptible with these individual inputs alone is profound.

Initially WIA was used as a research tool and as such has provided important mechanistic insights into a group of heterogeneous conditions all of which could be ultimately implemented clinically. Additionally, recognition of a cyclical portion of diastole in which are minimal identified a naturally “minimal resistance” period of the cardiac cycle leading to the development of the clinical tool of iFR.

This review article has highlighted current evidence but also suggests future avenues of research. Currently, coronary wave-intensity is hampered only by the need for invasive measurements of pressure and flow which provide some limitations to these applications. However, with improvements in technology, it is possible that coronary wave intensity could be moved out of the invasive catheter laboratory environment and into the non-invasive domain. This will allow larger studies to be undertaken, perhaps with sequential follow up in patients, enhancing the applicability of techniques. Ultimately, it may be used for prognostication in a variety of conditions including hypertension, HCM, aortic stenosis and heart failure.
